# The role of breastfeeding, antibiotics and antimalarial medicinal exposure in paediatric depression amongst children aged under 5: a population-based study

**DOI:** 10.1186/s12887-024-05265-1

**Published:** 2025-05-17

**Authors:** Kanayo Umeh, S Adaji

**Affiliations:** 1 School of Psychology, Faculty of Health, Liverpool John Moores University, Liverpool, Merseyside, L3 3AF UK; 2 Sessional General Practitioner, Bousfield Health Centre, Westminster Road, Liverpool, L4 4PP UK

## Abstract

**Background:**

Although paediatric depression is a recognised problem worldwide, there is limited data on its prevalence in children aged under 5 years, and the role of breastfeeding and medicinal exposure. This study examined whether lack of breastfeeding, and exposure to antimalarial and antibiotic medicines, contribute to depression in Nigerian children aged under 5.

**Methods:**

The study examined data from 31,103 children (mean age, 2.11 years (SD, 1.421)), collected during the 6th round of the MICS (Multiple Indicator Cluster Surveys), completed in 2021. A series of binary logistic regression models were used to analyse the data.

**Results:**

Children exposed to antimalarial medicines, specifically ACT (AOR = 1.760, 95%CI [1.316–2.355]), and artesunate injections (AOR = 1.757, 95%CI [1.150–2.684]), and those who were not breastfed (AOR = 0.776, 95%CI [0.625–0.963]), had greater odds of depression, after adjusting for potential confounders. The association between ACT medication use and depression was salient in male children (AOR = 2.347, 95%CI [1.547–3.559]), whereas the relationship between breastfeeding and depression was primarily observed in female children (AOR = 0.689, 95%CI [0.498–0.951]). Robustness checks including variations in managing missing data, breastfeeding measurement, and analysis across subgroups for multiple covariates (e.g., geographical area, mothers’ education, wealth index, medical symptoms), highlighted the importance of contextual factors.

**Conclusions:**

This is the first population-based study to examine the role of breastfeeding and medicinal use in suspected depression amongst children aged under 5, from sub-Sharan Africa. Overall, exposure to antimalaria treatment, particularly ACT, and inadequate breastfeeding may help identify young children susceptible to paediatric depression.

**Clinical trial number:**

Not applicable.

## Background

### Childhood depression in Sub-Saharan Africa

Although paediatric depression is a growing public health concern worldwide (Ghandour et al. 2019; Mullen 2018; Patra 2019), there has been limited research on the scope of the problem and its associated factors, especially in sub-Saharan Africa (Tomlinson and Morgan 2015). One reason for the limited evidence base is that diagnosing depression in children, particularly very young ones (e.g., under 5s), can be challenging (Alsaad et al. 2022; Patra 2019). Symptom presentation is complicated by age, and emotional, cognitive, and biological complexities (Selph and McDonagh 2019). Children under 5 are unable to verbalise being depressed, and/or may not meet clinical diagnostic criteria (e.g., Diagnostic and Statistical Manual for Mental Disorders (DSM-5) (Mullen 2018). Research suggests that, for very young children, researchers rely on parental reports for detecting and/or managing mood disorders in children (Reardon et al. 2017).

There has been no systematic review of research on childhood depression in sub-Saharan Africa over the past decade. The most recent data suggests 14.3% of children aged 0 to 16 years are living with mental health problems, including mood disorders (Cortina et al. 2012). The limited available evidence is almost entirely based on general populations (Gbadamosi et al. 2022; Yohani et al. 2023), adolescents (Sequeira et al. 2022; Partap et al. 2023; Aluh et al. 2018; Jorns-Presentati et al. 2021; Mabrouk et al. 2022), mixed populations (Owen et al. 2016), children aged over 5 years (Sherr et al. 2020; Mokwena et al. 2023), or parents/caretakers (Motlhatlhedi et al. 2017; Huang et al. 2017; Saeed and Wemakor 2019). There is almost no data on paediatric depression in children aged *under* 5 years, from sub-Saharan Africa (Tomlinson and Morgan 2015). Epidemiological evidence specifically on prevalence rates and/or causal factors in this age-group is extremely rare (Owen et al. 2016). This paucity of data is problematic because young children in sub-Saharan Africa face mental health challenges aggravated by chronic poverty, traumatic childhood events (e.g., witnessing violence), and illiteracy, compounded by parental difficulties, such as maternal depression (Tomlinson and Morgan 2015; Kurtz et al. 2023; Amene et al. 2024; Jorns-Presentati et al. 2021).

While a few studies from southern Africa have published data on depression in very young children (Mokwena et al. 2023; Sherr et al. 2020; Huang et al. 2017), including under 5’s (Drago et al. 2020; Motlhatlhedi et al. 2017), evidence from West African countries is sparse. Thus, there is little or no understanding of the prevalence of childhood depression and/or its underlying factors in the latter region. Rarely explored factors that may contribute to depression in West African children include medicinal use, particularly antimalarial (Nevin and Croft 2016) and antimicrobial (i.e., antibiotic) medication (Dinan and Dinan 2022), and breastfeeding history, which have been subjects of much debates.

### Overall objective

Although previous studies have assessed paediatric depression in very young children from sub-Saharan Africa, including under 5’s, most of this evidence is from southern Africa (Mokwena et al. 2023; Sherr et al. 2020; Huang et al. 2017; Drago et al. 2020; Motlhatlhedi et al. 2017). Data from West African countries is more difficult to find, despite the high rates of antimalarial and antibiotic consumption in West African children (Efunshile et al. 2019; Auta et al. 2019; Hossain et al. 2023a), lower breast-feeding rates in the region (Issaka et al. 2017), and evidence implicating antimalarial/antibiotic drugs and breastfeeding in childhood depression (Bitta et al. 2017; Nevin and Croft 2016; Lavebratt et al. 2019; Prichett et al. 2022). To address this gap in the literature, as well as generate a body of information that would improve understanding of childhood depression, the current study analysed survey data from 30,804 Nigerian children aged under 5.

The overall objective of this study was to explore the role of breastfeeding and commonly prescribed antimalarial and antimicrobial medicines in suspected depression amongst children aged under 5. Given a contemporary focus on malaria in the context of nutrition amongst children aged under 5 (Sarfo et al. 2023), we first report the general methodology, then present the background research and key findings as two separate papers: the first (Paper 1) covers the role of antimalarial medicines and breastfeeding in childhood depression, while the second (Paper 2) examines the contribution of antibiotics.

## General methodology

This study analysed data from the 2021 MICS/NICS conducted in Nigeria. The survey was funded by the Nigerian government, UNICEF, GAVI, and BMGF, and implemented by NBS, with technical support from UNICEF. Detailed and comprehensive information about MICS/NICS history in Nigeria, and organisation, methodology, sampling and implementation of the 2021 survey in the region, including personnel involved and questionnaires used, have been published elsewhere (MICS 2022). For the purposes of this paper, we will report only information relevant to the current objectives. The MICS survey collected data on socio-demographic and socio-economic characteristics (e.g., household, and personal assets), and multiple health-related factors concerning women and children, including post-natal care, disease episodes such as diarrhoea and malaria, treatment of children with fever, antimalarial treatment, use of antibiotics, young child feeding including breastfeeding, and equity-related topics, such as child functioning and subjective well-being.

Data collection stages are summarised in Fig. 1. Data was collected using five questionnaires, including a household survey on basic demographic information, a survey administered to all women in each household aged 15 to 49 years, and an under 5 questionnaire completed by mothers or caretakers of all children aged below 5 years, living in each household. Although the questions were based on standard MICS6 measures (MICS 2022), they were pre-tested and customised in several regions (states) of Nigeria during July 2021. Data collection involved pre-tested computer-assisted personal interviewing (CAPI), whereby a respondent or interviewer uses an electronic device (tablet computers, running the Window 10 operating system), to answer the questions. The interviews and questionnaires were translated into English, or the respondent’s native language (e.g., Hausa, Igbo, Fulani), based on literacy testing. Data collection occurred from September to December 2021 and was conducted by 74 teams of interviewers (MICS 2022).

### Data analyses

Chi-square and independent samples t-tests were used to analyse descriptive data. For hypotheses testing we conducted hierarchical binary logistic regression analyses. The overall sample size (*N* = 31103) provided sufficient statistical power to detect significant associations, reducing type II error rates.

To test the assumption of linearity in the logit, whereby the log-odds of the predicted probabilities of the outcome (depression) is a linear function of three *continuous* independent variables (age, weight, and wealth index), we conducted a Box-Tidwell test. This required adding a product term between each predictor and its natural log transformation, into the logistic regression model and examining its significance (Harris 2021).

We observed significant coefficients for all three interaction terms, suggesting the assumption of linearity in the logit was violated. However, due to the very large sample size, we decided to create and inspect scatterplots with the continuous predictors on the *x*-axis and the log-odds of the predicted probabilities on the *y*-axis. The observed curve estimates indicated a good fit for both linear and non-linear relationships. For the purposes of this investigation, we assumed linearity of the continuous predictors and log odds.

The outcome variable in logistic regression analyses was childhood depression — 1 for presence and 0 for absence. Predictor variables comprised exposure to breastfeeding, eight antimalarial medicines (ACT, fansidar, chloroquine, amodiaquine, quinine pills, quinine injection, artesunate rectal, and artesunate injection), and three types of antibiotic drugs (amoxicillin, cotrimoxazole, herbal). We adjusted for ten covariates (age, gender, weight, urban/rural area, mothers’ education, child’s functional difficulties, ethnicity/tribe, wealth index, and fever/cough symptoms. Three logistic regression models were tested: Model 1 (childhood depression = Intercept + medication exposure), Model 2 (childhood depression = Intercept + medication exposure + breastfeeding exposure), Model 3 (childhood depression = Intercept + medication exposure + breastfeeding exposure + covariates). Of particular interest was whether any significant association of childhood depression with medication and breastfeeding (Model’s 1 and 2) persisted after accounting for covariates (Model 3). The analysis was first conducted for the whole sample and repeated separately for males and females. Further analyses were then performed to assess the robustness of the findings across various sources of uncertainty.

## Paper 1: the role of breastfeeding and antimalarial medicines

### Introduction

Malaria infestation is a common cause of febrile illness among children in sub-Saharan Africa (Okiro and Snow 2010; Trape et al. 2023; Oshagbemi et al. 2023). Malarial treatment among Nigerian children, as in adults, follows WHO guidelines with recommendation for artemisinin-based combination therapy (ACT) (FMoH 2015). Among under-5 Nigerian children, there has been incremental resort to use ACT; from 7% in 2008 when the ACT was introduced to 74% in 2021 (NMEP 2022). Hossain et al. recently reviewed Malaria Survey datasets from 19 low- and middle-income countries (LMIC) and found that about one-fifth of Nigerian children under – 5 were suspected to have received antimalarial treatment (Hossain et al. 2024).

Antimalarial drugs have been associated with mental and neurological dysfunction (Bitta et al. 2017; Nevin and Croft 2016; Meier et al. 2004). For example, antimalarial medicines (e.g., chloroquine) may produce a wide spectrum of psychopathological experiences, including mood disorders (Maxwell et al. 2015). Although the psychiatric effects of antimalarial drugs in young children are complex and not well understood (Nevin and Croft 2016), the term ‘cerebral malaria’ is a recognised neurological complication in children aged under 5 years, which entails a history of fever (2–3 days), and may include cognitive dysfunction (Song et al. 2022).

Children aged under 5 account for two-thirds of all malaria deaths in sub-Saharan Africa (Sarfo et al. 2023). Thus, unsurprisingly, use of antimalarial medicines during pregnancy is common in the region (Nyeko et al. 2023; Hossain et al. 2023a). An analysis of cross-sectional data from 32,397 children aged under 5, from five malaria-endemic African countries, including Guinea, Mali, Nigeria, and Senegal, revealed anti-malarial consumption rates as high as 66.3%, (Guinea), 70.5% (Mali), and 31.6% (Nigeria), over the 2-week period prior to the survey (Hossain et al. 2023a). However, the psychiatric aspects of antimalarial drugs in very young children is not well understood (Aneja et al. 2019). Hitherto, most studies on the neurological effects of antimalarial medicines have focused on adult or mixed populations outside sub-Saharan Africa (Meier et al. 2004; Bitta et al. 2017; Nevin and Croft 2016; Maxwell et al. 2015; Song et al. 2022).

Meta–analytic evidence from 29 sub-Saharan African countries suggests lower breast-feeding rates (both early initiation, and exclusive breastfeeding) in West and Central African countries (Issaka et al. 2017). Breastfeeding levels in these regions were below the WHO’s recommended 50% (Zong et al. 2021). Regardless, data on the association between breastfeeding and paediatric depression is ambiguous: a systematic review of 21 studies on the effects of breastfeeding on mental health in mothers and children, including five studies of childhood depressive disorders, found conflicting evidence (Bugaeva et al. 2023).

While some research found no link between breastfeeding and childhood depression, after adjusting for multiple covariates (Kwok et al. 2013; Loret de Mola et al. 2016), other evidence suggested a modest protective effect for breastfeeding (i.e., lack of breastfeeding was associated with a higher likelihood of paediatric depression later in life) (Zhong et al. 2013; Allen et al. 1998). Other research suggests breastfeeding improves cognitive and brain functioning in children, partly due to fatty acids present in breastmilk (Krol and Grossmann 2018).

Overall, data from sub-Sahara Africa is extremely limited, and more research is needed to better understand how inadequate breastfeeding relates to psychiatric disorders in very young children from this region.

### Objective

Paper 1 explores the role of breastfeeding and commonly prescribed antimalarial medicines in suspected depression amongst children aged under 5. We hypothesised that children suspected to have depression are less likely to have been breastfed, and more likely to have received antimalarial medication, consistent with previous research.

### Method

#### Sample

The initial target sample size for the MICS was 37,000 households, covering 36 states, the Federal Capital Territory (FCT), and six geo-political zones in Nigeria. Within each region, a household listing was first generated in a selected number of census enumeration areas. A systematic sample of 20 households was then selected in each area. In total, 31,103 children aged under 5 years participated. Of this figure, more than half were male (50.4%), aged 24 to 59 months (two to five years) (62.4%), and lived in a rural area (63.4%). For most children (94.8%) their mother was the respondent to the under – 5 questionnaire/interview, while a primary caretaker responding on behalf of the other children. Most of the mothers either lacked any formal education, or had only primary school education (55.2%), and almost all the children (97%) lacked health insurance coverage.

#### Measures

##### Exposure to antimalarial drugs

Medicinal use was assessed using a series of multiple-choice questions, under a section labelled ‘Care of Illness’ (MICS 2022). This section focused on the type and/or source of advice or treatment provided in response to childhood illnesses during the past fortnight, notably diarrhoea (‘In the last two weeks, has [name] had diarrhoea’? ‘Yes’/‘No’/‘Don’t know’), fever (‘At any time in the last two weeks, has [name] been ill with a fever’? ‘Yes’/‘No’/‘Don’t know’), and cough (‘At any time in the last two weeks, has [name] had an illness with a cough’? ‘Yes’/‘No’/‘Don’t know’).

Several questions were used: ‘At any time during the illness, was [name] given any medicine for the illness? (‘Yes’/‘No’/‘Don’t know’), ‘What medicine was [name] given?’, ‘Any other medicine? The researcher recorded all medicines mentioned. If the type of medicine given could not be determined from a participant’s response, the researcher noted the brand name and then temporarily record ‘W’ until the appropriate category of medicine could be established.

Nine categories of antimalarial medicines were recorded: artemisinin combination therapy (ACT) (coded A), SP/fansidar (coded B), chloroquine (coded C), amodiaquine (coded D), quinine pills (coded E), quinine injection/IV (coded F), artesunate rectal (coded G), artesunate injection/IV (coded H), other anti-malarial (coded K). Due to its unidentified label, we excluded the last category from further analysis.

##### Breastfeeding

WHO and UNICEF guidelines both recommend exclusive breastfeeding for the first six months of life, and breastfeeding up to the age of 2 years, and beyond (Aryeetey and Dykes 2018). MICS literature considers a broad range of multiple breastfeeding metrics (MICS 2022). However, given the lower breast-feeding rates (both early initiation, and exclusive breastfeeding) in West and Central African countries (Issaka et al. 2017), for the purposes of this study we focused primarily on overall breast feeding history (i.e., prior exposure to breastfeeding). This was assessed using two items contained within the ‘breastfeeding and dietary’ intake section of the MICS questionnaire for children aged under 5: ‘Has [name] ever been breastfed?’ ‘Yes’ (coded 1)/ ‘No’ or ‘don’t know’ (coded 0), and ‘Is [name] still being breastfed?’ ‘Yes’ (coded 1)/ ‘No’ or ‘don’t know’ (coded 0).

##### Childhood depression

Children aged under 5 are generally unable to verbalise being depressed, and hence may not meet the clinical diagnostic criteria (e.g., Diagnostic and Statistical Manual for Mental Disorders (DSM-5) for depression (Mullen 2018). Consequently, research has generally relied on parental feedback (Reardon et al. 2017). Childhood ‘depression’ or ‘sadness’ in the MICS was assessed with a single question (MICS 2022). This item was part of series of multiple-choice questions assessing early childhood development, including linguistic acuity and interactions with other children and household members. The data collector made a specific statement, directed at the child’s mother or caretaker: ‘The next two questions have five different options for answers. I am going to read these to you after each the question. How often does [name] seem to be very sad or depressed? Would you say: ‘daily’ (coded 1), ‘weekly’ (coded 2), ‘monthly’ (coded 3), ‘a few times a year’ (coded 4) or ‘never’ (coded 5)’? Depending on participant feedback the researcher could record two additional response options: ‘don’t know’ (coded 8) or ‘no response’ (coded 9).

For the purposes of subsequent binary logistic regression analysis, we collapsed these response options into a dichotomous variable: ‘depressed’ (incorporating ‘daily’, ‘weekly’, ‘monthly’, ‘a few times a year’) (coded 1) and ‘never’ (comprising ‘never’ and the other response options) (coded 0).

##### Confounding factors

We examined a total of ten covariates: (a) gender (‘male’ (coded 1) / ‘female’ (coded 2)), (b) age, (c) weight, (d) geographical area ( ‘urban’ (coded 1) / ‘rural’ (coded 2)), (e) mother’s or caretaker’s educational level ( ‘none’ (coded 0), ‘primary’/ ‘secondary’/ ‘higher’ (coded 1)), (f) child’s functional difficulties (CFD), (g) ethnicity/tribe of the head of the household (‘Hausa’/ ‘Igbo’/ ‘Yoruba’/ ‘Fulani’ (coded 1), ‘others’ (coded 0)), (h) wealth index, (i) fever symptoms (‘Yes’ (coded 1)/ ‘No’ (coded 0)) and (j) cough symptoms (‘Yes’ (coded 1)/ ‘No’ (coded 0)).

Age was based on birth history information, and recorded in days, months, and years. For the purposes of this paper, we analysed number of days. Weight was calibrated in kilogrammes (kg), and based on measurements of each child, read out by a measurer. CFD incorporated questions about physical difficulties that a child may have including hearing (e.g., ‘Does [name] use a hearing aid?’), vision (e.g., ‘Does [name] wear glasses?’), and mobility (e.g., ‘Does [name] use any equipment or receive assistance for walking?’). CFD data was only collected for children aged 2 to 4 years.

Wealth index is a pre-computed composite numerical MICS metric, reflecting wealth or assets in each household (MICS 2022). It incorporates various household characteristics, including ownership of personal assets (e.g., refrigerator, television, air conditioner, fan, water heater, bicycle, car, truck or van), and access to basic services (e.g., electricity, energy for cooking, internet) (Xie et al. 2023). Scores are organised into five wealth index quintiles: ‘poorest’ (coded 1), ‘second’ (coded 2), ‘middle’ (coded 3), ‘fourth’ (coded 4),‘richest’ (coded 5).

Finally, we deemed it essential to account for fever and cough symptoms, as covariates. High fever in children is associated with malaria (El-Radhi 2019), and a cough may be caused by pneumonia but also by severe malaria (Ashley et al. 2018), triggering the use of antimalarial medicines.

### Results

#### Descriptive data

Rates of missing data ranged from 0 (e.g., medication use, cough/fever symptoms) to 37.2% (child functional difficulties), depression (46.2%), and 51.5% (cough symptoms). We opted for listwise deletion of missing cases (Schober and Vetter 2020), given the large overall sample size (and hence statistical power) (Serdar et al. 2021). However, the expected maximisation (EM) method, which replaces missing values with estimated parameters (Stavseth et al. 2019), was later used for sensitivity analysis (robustness checks).

Descriptive characteristics for the whole sample, and children with and without depression, is shown in Table 1. Overall, 9774 children (31.4%) were suspected to have depression. Depressed children were more likely than their non-depressed counterparts to live in a rural area (75.3% versus 70.9%, respectively), (χ^2^ (1, *N* = 16720) = 40.293, *p* < 0.001), less likely to have an educated mother (52.3% versus 60%, respectively), (χ^2^ (1, *N* = 16718) = 97.570, *p* < 0.001), more likely to have functional difficulties (5.2% versus 3.3%, respectively), (χ^2^ (1, *N* = 16720) = 35.140, *p* < 0.001), experience fever (26.2% versus 22.1%, respectively), (χ^2^ (1, *N* = 16720) = 35.725, *p* < 0.001) and cough (19.1% versus 15.6%, respectively), (χ^2^ (1, *N* = 16720) = 34.028, *p* < 0.001), have lower body weight (M = 0.932 versus 1.120, respectively) (*t*(14708.053) = −12.011, *p* < 0.001), and live in a less wealthy household (M = 2.45 versus 2.61, respectively) (*t*(14324.925) = −7.613, *p* < 0.001).

**Table 1:** Descriptive data for study variables

**Variables**	**Whole sample**	**Childhood depression **		***P***
Antibiotics		**No**	**Yes**	
Amoxicillin	471 (2.8%)	180 (2.6%)	291 (3%)	χ^2^(1, *N* = 16720) = 2.208, *p* > 0.05
Cotrimoxazole	139 (0.8%)	43 (0.6%)	96 (1.0%)	χ^2^(1, *N* = 16720) = 6.495, *p* < 0.01
Herbal	241 (1.4%)	93 (1.3%)	148 (1.5%)	χ^2^(1, *N* = 16720) = 0.879, *p* > 0.05
Antimalarials				
ACT	810 (4.8%)	272 (3.9%)	538 (5.5%)	χ^2^(1, *N* = 16720) = 22.225, *p* < 0.001
Fansidar	165 (1.0%)	52 (0.7%)	113 (1.2%)	χ^2^(1, *N* = 16720) = 6.900, *p* < 0.01
Chloroquine	234 (1.4%)	71 (1.0%)	163 (1.7%)	χ^2^(1, *N* = 16720) = 12.261, *p* < 0.001
Amodiaquine	124 (0.7%)	45 (0.6%)	79 (0.8%)	χ^2^(1, *N* = 16720) = 1.419, *p* > 0.05
Quinine (pills)	74 (0.4%)	23 (0.3%)	51 (0.5%)	χ^2^(1, *N* = 16720) = 3.350, *p* < 0.05
Quinine (injection)	229 (1.4%)	71 (1.0%)	158 (1.6%)	χ^2^(1, *N* = 16720) = 10.619, *p* < 0.001
Artesunate (rectal)	150 (0.9%)	60 (0.9%)	90 (0.9%)	χ^2^(1, *N* = 16720) = 0.148, *p* > 0.05
Artesunate (injection)	382 (2.3%)	129 (1.9%)	253 (2.6%)	χ^2^(1, *N* = 16720) = 9.727, *p* = 0.001
Breastfeeding (ever)	4864 (92.6%)	2238 (93.8%)	2626 (91.6%)	χ^2^(1, *N* = 5253) = 9.218, *p* = 0.001
Covariates				
Age (number of days)	M, 975.11 (SD: 790.015 )	M,1312.71 (SD: 782.450)	M,1331.35 (SD: 667.279)	*t*(13429.854) = 1.612, *p* > .05
Gender (% Female)	8231 (49.2%)	3449 (49.7%)	4782 (48.9%)	χ^2^(1, *N* = 16720) = 0.863, *p* > 0.05
Weight (kg) (MICS + NICS)	M, 0.990 (SD: 0.959)	M, 1.120 (SD:1.00)	M, 0.932 (SD:0.978)	*t*(14708.053) = −12.011, *p* < .001
Area (% rural)	12285 (73.5%)	4925 (70.9%)	7360 (75.3%)	χ^2^(1, *N* = 16720) = 40.293, *p* < 0.001
Education (% educated)	9273 (55.5%)	4165 (60.0%)	5108 (52.3%)	χ^2^(1, *N* = 16718) = 97.570, *p* < 0.001
Functional difficulties	738 (4.4%)	229 (3.3%)	509 (5.2%)	χ^2^(1, *N* = 16720) = 35.140, *p* < 0.001
Ethnicity/tribe (%)	10189 (60.9%)	4253 (61.2%)	5936 (60.7%)	χ^2^(1, *N* = 16720) = 0.421, *p* > 0.05
Wealth index (HH assets)	M, 2.56 (SD: 1.349)	M, 2.61 (SD:1.365)	M, 2.45 (SD:1.276)	*t*(14324.925) = −7.613, *p* < .001
Illness symptoms (fever)	4094 (24.5%)	1537 (22.1%)	2557 (26.2%)	χ^2^(1, *N* = 16720) = 35.725, *p* < 0.001
Illness symptoms (cough)	2958 (17.7%)	1087 (15.6%)	1871 (19.1%)	χ^2^(1, *N* = 16720) = 34.028, *p* < 0.001

*Notes.* *NR *Non-response, *HH *Household. All values are sample size and percentages (%), unless means (M) and standard deviations (SDs) are stated. Percentages for all medicines depict the proportion of children exposed to each drug. Percentage for breastfeeding indicates the proportion of children who had ever been breastfed. Percentage for education (maternal) refers to the proportion with primary, secondary, or higher education. Percentage for ethnicity/tribe depicts proportion of children from Hausa, Igbo, or Yoruba backgrounds. *P* values relate to 1-sided comparisons between depressed and non-depressed children, based on Chi-square or independent samples t-tests

Depressed children were more like than non-depressed participants to be administered ACT (5.5% versus 3.9%, respectively), (χ^2^ (1, *N* = 16720) = 22.225, *p* ≤ 0.001), fansidar (1.2% versus 0.7%, respectively), (χ^2^ (1, *N* = 16720) = 6.900, *p* ≤ 0.01), chloroquine (1.7% versus 1%, respectively), (χ^2^ (1, *N* = 16720) = 12.261, *p* ≤ 0.001), quinine (pills) (0.5% versus 0.3%, respectively), (χ^2^ (1, *N* = 16720) = 3.350, *p* < 0.05), quinine (injection) (1.6% versus 1%, respectively), (χ^2^ (1, *N* = 16720) = 10.619, *p* < 0.001), and artesunate (injection) (2.6% versus 1.9%, respectively), (χ^2^ (1, *N* = 16720) = 9.727, *p* ≤ 0.001). By contrast, depressed children were less likely to have been breastfed (91.6% versus 93.8%, respectively), (χ^2^ (1, *N* = 5253) = 9.218, *p* ≤ 0.001). We observed no group differences based on gender, age, ethnicity/tribe, and consumption of amodiaquine, and artesunate (rectal).

Overall, most children were male (50.8%), lived in a rural area (73.5%), had an educated mother (55.5%), did not suffer functional difficulties (95.6%), belonged to the Hausa, Igbo, Yoruba, or Fulani tribe (60.9%), and had not experienced a fever (75.5%) or cough (82.3%). The average age (in days), body weight (kg), and wealth index were 975.11 (SD = 790.015), 0.990 (SD = 0.959), and 2.56 (SD = 1.349), respectively (the average age equates to 32.055 months). Only a small minority of children (≤ 4.8%) had been exposed to any medicines. However, most (92.2%) had been breastfed (25.7% were still breastfeeding). Breastfeeding children were significantly younger than their non-breastfeeding counterparts (mean age 348.42 days [0.954 years]) versus 1194.67 days [3.273 years] respectively, (*t*(17890.621) = −105.279, *p* < 0.001).

#### Predicting childhood depression in the whole sample

Table 2 shows logistic regression estimates, together with overall model parameters. Exposure to ACT, (OR = 1.647, 95%CI [1.262–2.150]), chloroquine, (OR = 1.632, 95%CI [1.022 − 2.604]), and artesunate injections (OR = 1.710, 95%CI [1.137 − 2.573]), predicted higher odds of childhood depression (Model 1) (Fig. 2). Thus, for children administered ACT, chloroquine, and artesunate treatment, there was a significant 1.647, 1.632, and 1.710 increase, respectively, in the odds of experiencing depression, compared to children not given these medicines. Addition of breastfeeding (Model 2) also resulted in good fit: previous breastfeeding was associated with lower odds of depression (OR = 0.719, 95%CI [0.581–0.890]). This adjusted model was significant (χ²(1) = 46.854, *p* < 0.001), showing that ACT (AOR = 1.650, 95%CI [1.264 − 2.153]), chloroquine (AOR = 1.612, 95%CI [1.009 − 2.574]), artesunate injection (AOR = 1.726, 95%CI [1.147 − 2.598]), and breastfeeding exposure (OR = 0.719, 95%CI [0.581–0.890]), robustly predict childhood depression. The model’s pseudo-R² value of 0.012 (Nagelkerke) indicated improvement over the null model. The contributions of ACT, artesunate injection, and breastfeeding to the model remained significant even after adjusting for covariates (Model 3), with the odds of depression higher in children exposed to ACT (OR = 1.760, 95%CI [1.316–2.355]) and artesunate injections (AOR = 1.757, 95%CI [1.150 − 2.684]), and lower in breastfed children (AOR = 0.776, 95%CI [0.625 − 0.963]).

**Table 2:** Hierarchical logistic regression of predictors of paediatric depression in all infants and children

	Model 1		Model 2		Model 3	
Predictors	ß	Exp(ß) (95% CI)	ß	Exp(ß) (95% CI)	ß	Exp(ß) (95% CI)
Antibiotics (Amoxicillin)	−0.003	0.997 (0.719, 1.382)	−0.008	0.992 (0.715, 1.375)	0.000	1.019 (0.727, 1.427)
Antibiotics (Cotrimoxaz)	0.595	1.812 (0.956, 3.435)	0.605	1.831 (0.966, 3.471)	0.444	1.559 (0.814, 2.988)
Antibiotics (Herbal)	0.020	1.020 (0.654, 1.589)	0.015	1.015 (0.651, 1.583)	−0.058	0.944 (0.597, 1.492)
Antimalaria (ACT)	0.499^c^	1.647 (1.262, 2.150)	0.501^c^	1.650 (1.264, 2.153)	0.565^c^	1.760 (1.316, 2.355)
Antimalaria (Fansidar)	0.047	1.048 (0.606, 1.813)	0.026	1.026 (0.592, 1.777)	0.008	1.008 (0.575, 1.770)
Antimalaria (Chloroquine)	0.490^a^	1.632 (1.022, 2.604)	0.477^a^	1.612 (1.009, 2.574)	0.460	1.584 (0.982, 2.556)
Antimalaria (Amodiaquine)	0.411	1.508 (0.720, 3.157)	0.416	1.516 (0.724, 3.176)	0.569	1.766 (0.834, 3.738)
Antimalaria (Quinine/pills)	0.601	1.824 (0.787, 4.227)	0.588	1.801 (0.777, 4.177)	0.649	1.914 (0.815, 4.494)
Antimalaria (Quinine/inject)	−0.307	0.736 (0.449, 1.205)	−0.296	0.744 (0.454, 1.219)	−0.255	0.775 (0.467, 1.285)
Antimalaria (Artesunate/rectal)	0.275	1.316 (0.686, 2.523)	0.292	1.339 (0.698, 2.569)	0.420	1.522 (0.779, 2.973)
Antimalaria (Artesunate/inject)	0.536^b^	1.710 (1.137, 2.573)	0.546^b^	1.726 (1.147, 2.598)	0.563^b^	1.757 (1.150, 2.684)
Breastfeeding (ever)			−0.330^b^	0.719 (0.581, 0.890)	−0.254^a^	0.776 (0.625, 0.963)
Age (in days)					0.000	1.000 (1.000, 1.000)
Gender (M/F)					−0.011	0.989 (0.886, 1.105)
Weight (MICS/NICS)					0.200^c^	0.819 (0.768, 0.873)
Area (urban/rural)					0.055	1.057 (0.910, 1.227)
Education (mothers)					−0.303^c^	0.738 (0.649, 0.840)
Childs functional difficulties					0.366^b^	1.442 (1.146, 1.814)
Ethnicity(tribe)					0.071	1.074 (0.954, 1.209)
Wealth index					−0.028	0.972 (0.920, 1.027)
Medical (fever)					−0.132	0.876 (0.746, 1.030)
Medical (cough)					0.213^b^	1.238 (1.067, 1.436)
Nagelkerke *R* ^*2*^	0.010		0.012		0.040	
Cox & Snell *R* ^*2*^	0.007		0.009		0.030	
−2 Log likelihood	7199.049		7189.666		7076.719	
No. of observations	31,103		31,103		31,103	
Model Chi-square	37.471^c^		46.854^c^		159.801^c^	

Note. Coding: Unless otherwise indicated, all predictor variables except for age, weight, and wealth index, are dummy variables, coded as ‘Yes’ (1), ‘No’ (0)). Gender is coded as ‘Male’ (1), ‘Female’ (0). Geographical area is coded as ‘Urban’ (1) vs ‘Rural’ (0). Education (mothers) is coded as ‘None’ (0), ‘Primary’/‘Secondary’/‘Higher’ (1). Ethnicity (of household head) is coded as ‘Hausa’/‘Igbo’/‘Yoruba’/‘Fulani’ (1), ‘Other’ (0). ACT = Artemisinin-based combination therapy; MICS = Multiple Indicator Cluster Surveys; NICS = National Immunization Coverage Survey. ‘No’ includes ‘don’t know’ and no response. ^a^ *p* ≤ 0.05, ^b^ *p* ≤ 0.01, ^c^ *p* ≤ 0.001,

#### Predicting childhood depression in males

Table 3 shows logit estimates and model parameters for male children. Exposure to ACT (OR = 2.188, 95%CI [1.490–3.212]) and artesunate injections (OR = 1.770, 95%CI [1.020–3.071]) was associated with a greater risk of childhood depression (Model 1). Male children given ACT were two times more likely to experience depression, while those receiving artesunate injections were 1.770 times more prone to depression, compared to male children who did not receive these treatments (χ²(1) = 32.023, *p* < 0.001, pseudo-R² = 0.016). Inclusion of breastfeeding (Model 2) failed to improve model fit. Addition of covariates (Model 3) generated a significant model (χ²(1) = 92.581, *p* < 0.001), negating the contribution of artesunate exposure, albeit ACT remained significant: male children administered ACT were more than twice as likely to be depressed compared to those not given this treatment (AOR = 2.347, 95%CI [1.547–3.559]).

**Table 3:** Hierarchical logistic regression of predictors of paediatric depression in male infants and children

	Model 1		Model 2		Model 3	
Predictors	ß	Exp(ß) (95% CI)	ß	Exp(ß) (95% CI)	ß	Exp(ß) (95% CI)
Antibiotics (Amoxicillin)	−0.174	0.840 (0.526, 1.342)	−0.175	0.840 (0.526, 1.341)	−0.128	0.879 (0.544, 1.423)
Antibiotics (Cotrimoxaz)	0.342	1.408 (0.579, 3.423)	0.349	1.418 (0.583, 3.448)	0.236	1.266 (0.508, 3.157)
Antibiotics (Herbal)	0.462	1.587 (0.824, 3.054)	0.457	1.579 (0.820, 3.041)	0.371	1.449 (0.738, 2.846)
Antimalaria (ACT)	0.783^c^	2.188 (1.490, 3.212)	0.788^c^	2.199 (1.498, 3.229)	0.853^c^	2.347 (1.547, 3.559)
Antimalaria (Fansidar)	−0.180	0.835 (0.417, 1.674)	−0.198	0.820 (0.409, 1.645)	−0.159	0.853 (0.416, 1.746)
Antimalaria (Chloroquine)	0.536	1.709 (0.865, 3.377)	0.524	1.689 (0.854, 3.339)	0.514	1.672 (0.833, 3.354)
Antimalaria (Amodiaquine)	0.231	1.260 (0.351, 4.528)	0.226	1.254 (0.349, 4.508)	0.485	1.624 (0.444, 5.933)
Antimalaria (Quinine/pills)	0.422	1.525 (0.506, 4.598)	0.406	0.501 (0.497, 4.528)	0.448	1.565 (0.509, 4.812)
Antimalaria (Quinine/inject)	−0.239	0.788 (0.412, 1.504)	−0.231	0.793 (0.415, 1.516)	−0.192	0.826 (0.420, 1.622)
Antimalaria (Artesunate/rectal)	0.596	1.815 (0.769, 4.287)	0.607	1.834 (0.776, 4.335)	0.789	2.200 (0.914, 5.298)
Antimalaria (Artesunate/inject)	0.571^a^	1.770 (1.020, 3.071)	0.581^a^	1.788 (1.030, 3.105)	0.552	1.736 (0.977, 3.084)
Breastfeeding (ever)			−0.241	0.786 (0.589, 1.050)	−0.158	0.854 (0.637, 1.146)
Age (in days)					0.000	1.000 (1.000, 1.000)
Weight (MICS/NICS)					−0.183^c^	0.833 (0.762, 0.910)
Area (urban/rural)					0.053	1.055 (0.858, 1.296)
Education (mothers)					−0.332^c^	0.717 (0.599, 0.859)
Childs functional difficulties					0.314	1.369 (0.991, 1.892)
Ethnicity(tribe)					0.136	1.145 (0.970, 1.352)
Wealth index					−0.038	0.962 (0.892, 1.038)
Medical (fever)					−0.100	0.905 (0.719, 1.139)
Medical (cough)					0.068	1.070 (0.869, 1.318)
Nagelkerke *R* ^*2*^	0.016		0.017		0.045	
Cox & Snell *R* ^*2*^	0.012		0.013		0.034	
−2 Log likelihood	3682.286		3679.599		3621.728	
No. of observations	15,799		15,799		15,799	
Model Chi-square	32.023^c^		34.709^c^		92.581^c^	

Note. Coding: Unless otherwise indicated, all predictor variables except for age, weight, and wealth index, are dummy variables, coded as ‘Yes’ (1), ‘No’ (0)). Geographical area is coded as ‘Urban’ (1) vs ‘Rural’ (0). Education (mothers) is coded as ‘None’ (0), ‘Primary’/‘Secondary’/‘Higher’ (1). Ethnicity (of household head) is coded as ‘Hausa’/‘Igbo’/‘Yoruba’/‘Fulani’ (1), ‘Other’ (0). ACT = Artemisinin-based combination therapy; MICS = Multiple Indicator Cluster Surveys; NICS = National Immunization Coverage Survey. ‘No’ includes ‘don’t know’ and no response. ^a^ *p* ≤ 0.05, ^b^ *p* ≤ 0.01, ^c^ *p* ≤ 0.001,

#### Predicting childhood depression in females

Table 4 depicts results for female children. The initial logistic regression model comprising medicinal exposure (Model 1) failed to fit the data well: none of the predictor variables was significant. Addition of breastfeeding (Model 2) significantly improved model fit (χ²(1) = 24.474, *p* < 0.05), with the pseudo-R² value of 0.013 (Nagelkerke) indicated a slight improvement over the null model. Female children who had been breastfed were less likely to experience depression, compared to those not breastfed (OR = 0.642, 95%CI [0.468–0.881]). Incorporating covariates (Model 3) improved model fit (χ²(1) = 86.606, *p* < 0.001), increasing the proportion of variance explained (pseudo-R² value of 0.045). Breastfeeding remained significant in the final model (AOR = 0.689, 95%CI [0.498–0.951]).

**Table 4:** Hierarchical logistic regression of predictors of paediatric depression in female infants and children

	Model 1		Model 2		Model 3	
Predictors	ß	Exp(ß) (95% CI)	ß	Exp(ß) (95% CI)	ß	Exp(ß) (95% CI)
Antibiotics (Amoxicillin)	0.099	1.104 (0.695, 1.752)	0.086	1.090 (0.686, 1.731)	0.075	1.078 (0.666, 1.745)
Antibiotics (Cotrimoxaz)	0.863	2.370 (0.927, 6.060)	0.878	2.407 (0.941, 6.159)	0.677	1.968 (0.760, 5.100)
Antibiotics (Herbal)	−0.369	0.691 (0.368, 1.298)	−0.373	0.689 (0.366, 1.294)	−0.457	0.633 (0.329, 1.217)
Antimalaria (ACT)	0.207	1.231 (0.842, 1.798)	0.199	1.220 (0.834, 1.783)	0.273	1.314 (0.864, 1.999)
Antimalaria (Fansidar)	0.399	1.491 (0.591, 3.757)	0.380	1.462 (0.578, 3.695)	0.249	1.282 (0.501, 3.282)
Antimalaria (Chloroquine)	0.435	1.546 (0.803, 2.976)	0.420	1.522 (0.789, 2.936)	0.367	1.443 (0.737, 2.828)
Antimalaria (Amodiaquine)	0.525	1.690 (0.680, 4.199)	0.541	1.717 (0.691, 4.270)	0.627	1.871 (0.740, 4.730)
Antimalaria (Quinine/pills)	0.803	2.233 (0.594, 8.398)	0.803	2.233 (0.593, 8.407)	0.956	2.602 (0.678, 9.985)
Antimalaria (Quinine/inject)	−0.349	0.705 (0.326, 1.528)	−0.330	0.719 (0.332, 1.558)	−0.270	0.764 (0.347, 1.682)
Antimalaria (Artesunate/rectal)	−0.072	0.930 (0.331, 2.616)	−0.040	0.961 (0.342, 2.702)	−0.038	0.963 (0.329, 2.822)
Antimalaria (Artesunate/inject)	0.437	1.548 (0.835, 2.869)	0.442	1.555 (0.839, 2.884)	0.511	1.667 (0.879, 3.161)
Breastfeeding (ever)			−0.443^b^	0.642 (0.468, 0.881)	−0.373^a^	0.689 (0.498, 0.951)
Age (in days)					0.000	1.000 (1.000, 1.000)
Weight (MICS/NICS)					−0.206^c^	0.813 (0.741, 0.893)
Area (urban/rural)					0.054	1.056 (0.850, 1.311)
Education (mothers)					−0.280^b^	0.756 (0.628, 0.911)
Childs functional difficulties					0.422	1.525 (1.099, 2.116)
Ethnicity(tribe)					0.006	1.006 (0.849, 1.193)
Wealth index					−0.021	0.979 (0.904, 1.062)
Medical (fever)					−0.154	0.857 (0.681, 1.078)
Medical (cough)					0.378^c^	1.460 (1.177, 1.810)
Nagelkerke *R* ^*2*^	0.009		0.013		0.045	
Cox & Snell *R* ^*2*^	0.007		0.010		0.033	
−2 Log likelihood	3505.412		3497.668		3435.536	
No. of observations	15,304		15,304		15,304	
Model Chi-square	16.730		24.474^a^		86.606^c^	

Note. Coding: Unless otherwise indicated, all predictor variables except for age, weight, and wealth index, are dummy variables, coded as ‘Yes’ (1), ‘No’ (0)). Geographical area is coded as ‘Urban’ (1) vs ‘Rural’ (0). Education (mothers) is coded as ‘None’ (0), ‘Primary’/‘Secondary’/‘Higher’ (1). Ethnicity (of household head) is coded as ‘Hausa’/‘Igbo’/‘Yoruba’/‘Fulani’ (1), ‘Other’ (0). ACT = Artemisinin-based combination therapy; MICS = Multiple Indicator Cluster Surveys; NICS = National Immunization Coverage Survey. ‘No’ includes ‘don’t know’ and no response. ^a^ *p* ≤ 0.05, ^b^ *p* ≤ 0.01, ^c^ *p* ≤ 0.001,

#### Robustness checks

We performed several robustness checks to determine how the results are affected by sources of uncertainty, including variations in analytic protocol, and management of missing data (Neumayer and Plumper 2017).

First, we repeated the logistic regression analysis using bootstrapping, whereby multiple (1000) simulated samples are tested. The emerging bootstrapped estimates supported the initial findings: exposure to ACT, (*β* = 0.565, 95%CI [0.268 to 0.857]), artesunate injections (*β* = 0.563, 95%CI [0.137 to 1.075]), and breastfeeding (*β* = −0.254, 95%CI [−0.478 to −0.044]), predicted childhood depression in the whole sample (final model). ACT exposure and breastfeeding remained salient predictors in males (*β* = 0.853, 95%CI [0.425 to 1.277]) and females (*β* = −0.373, 95%CI [−0.700 to −0.037]), respectively.

Next, we repeated the analyses using a different measure of breastfeeding (‘still’ breastfeeding rather than ‘ever’ breastfed). This produced partially identical results. In addition to ACT and artesunate (injection), exposure to chloroquine (AOR = 1.435, 95%CI [1.073–1.918]) and quinine (injection) (AOR = 1.372, 95%CI [1.026–1.836]) now predicted depression in the overall sample, helping improve model fit (χ²(22) = 357.907, *p* < 0.001). Breastfeeding was no longer significant. Analysis of the gender split showed that ACT exposure remained significant in males (AOR = 1.519, 95%CI [1.204–1.917]), whereas breastfeeding was no longer significant in females. Moreover, artesunate (injection) now improved model fit in the latter group (OR = 1.455, 95%CI [1.058–2.002]).

Third, we re-ran the analyses across age groups (≤ versus > 2 years). Medication use and child functional difficulties were excluded from this analysis, due to low cell frequencies. Breastfeeding exposure remained a significant predictor, but only in older children (i.e., those aged > 2 years) (AOR = 0.757, 95%CI [0.610–0.941]).

Next, we repeated the analyses across geographical (rural versus urban) regions. Antimalarial medicinal use remained a significant factor in both areas, with depression predicted by amodiaquine (AOR = 11.065, 95%CI [1.312–93.288]), and artesunate injection use (AOR = 0.757, 95%CI [0.610–0.941]) in urban children, and ACT exposure (AOR = 3.344, 95%CI [1.261–8.867]), in rural children. Breastfeeding was only significant in urban dwellers (AOR = 0.595, 95%CI [0.399–0.888]).

We then tested the hypotheses based on mother’s educational level (no education versus some education). ACT use predicted paediatric depression in non-educated mothers (AOR = 1.677, 95%CI [1.066–2.638]), while exposure to three medicines – ACT (AOR = 1.783, 95%CI [1.212–2.623]), chloroquine (AOR = 2.114, 95%CI [1.033–4.327]), and artesunate rectal exposure (AOR = 2.455, 95%CI [1.337–4.509]), were significant for children of educated mothers. Breastfeeding predicted depression solely in the educated group (AOR = 0.627, 95%CI [0.451–0.873]).

Next, we repeated the analyses by wealth index, comparing the lowest two wealth quintiles (‘poorest’, ‘second’) with the three higher categories (‘middle’, ‘fourth’, ‘richest’)). ACT exposure predicted depression in the less wealthy quintiles (AOR = 2.062, 95%CI [1.351–3.146]), whereas use of amodiaquine (AOR = 4.029, 95%CI [1.120–14.495]), and artesunate injections (AOR = 2.403, 95%CI [1.202–4.804]), were significant in children from wealthier backgrounds. Breastfeeding was not salient in either socio-economic group.

We re-ran the analyses as a function of medical symptoms (cough, fever). Regarding fever, antimalarial medicines – ACT (AOR = 1.695, 95%CI [1.259–2.281]) and artesunate injections (AOR = 1.602, 95%CI [1.044–2.457]) – were significant in febrile participants. Breastfeeding was only relevant in the former group (AOR = 0.732, 95%CI [0.569–0.941]). For cough symptoms, ACT (AOR = 1.732, 95%CI [1.179–2.544]) and chloroquine (AOR = 2.063, 95%CI [1.043–4.081]) predicted depression in asymptomatic children, while ACT (AOR = 1.809, 95%CI [1.150–2.846]) and artesunate injections (AOR = 1.602, 95%CI [1.044–2.457]) were significant in symptomatic children. Breastfeeding was only salient in the latter group (AOR = 0.599, 95%CI [0.361–0.994]).

Finally, we repeated the analyses using the EM approach for managing missing data. This produced roughly identical results with the original analyses (final model), whereby the odds of depression were elevated in children receiving ACT (AOR = 1.760, 95%CI [1.316–2.355]) and artesunate injections (AOR = 1.757, 95%CI [1.150 − 2.684]), and lower in breastfed children (AOR = 0.776, 95%CI [0.625 − 0.963]).

Overall, the pattern of results varied as a function of the type of breastfeeding measure used, contextual factors (e.g., age, geographical area, mother’s educational level, child functional difficulties, wealth index), and the method used to manage missing data. Nevertheless, we observed a recurring pattern, whereby antimalarial medicinal use (particularly ACT treatment and artesunate injections), and breastfeeding history were persistent correlates of 

## Discussion

Research on the psychiatric effects of antimalarial drugs in young children is very rare (Aneja et al. 2019), with most studies focusing on adults, or the general population (Meier et al. 2004; Bitta et al. 2017; Nevin and Croft 2016; Maxwell et al. 2015; Song et al. 2022). Thus, hitherto the association between antimalarial drugs and depression in children aged under 5 has been poorly understood (Aneja et al. 2019). Data for children from sub-Saharan Africa is particularly rare. Difficulty in identifying and diagnosing paediatric depression (Patra 2019), combined with the lack of research on childhood mental health in sub-Saharan Africa (Tomlinson and Morgan 2015) adds additional ambiguity.

Our findings suggest antimalarial treatment, and lack of breastfeeding may be independent risk factors for paediatric depression in Nigerian children aged under 5 years. While the psychiatric effects of antimalarial drugs have been attributed to neurological mechanisms, such as toxicity to the central nervous system (CNS) (Nevin and Croft 2016), an arguably more plausible explanation for the current findings is the emotional aspect of malaria symptoms (e.g., fever, headache, gastrointestinal effects) and/or antimalarial treatment (medicines, injection). These medical experiences can be very unpleasant, especially for very young children, who may express their discomfort in ways that denote ‘depression’ or ‘sadness’ to an adult observer (Annan et al. 2023). For example, antimalarial drugs can induce vomiting in and children (Creek et al. 2010). Injections are painful to very young children (Taddio et al. 2022), who often convey their distress through vocal/facial expressions denoting intense discomfort (Annan et al. 2023).

As hypothesised, breastfed children were less likely to be depressed, even after accounting for covariates. Although previous research has implicated inadequate breastfeeding in paediatric depression, the evidence has been mixed (Kwok et al. 2013; Loret de Mola et al. 2016; Allen et al. 1998). A systematic review and meta-analysis of 18 studies on breastfeeding and mental disorders found conflicting evidence on the protective effect of breastfeeding on children’s mental health in later life (Bugaeva et al. 2023). Some research found no association (Kwok et al. 2013; Loret de Mola et al. 2016), while other evidence indicated a protective effect for breastfeeding (Zhong et al. 2013; Allen et al. 1998). Our findings suggest a robust association between breastfeeding and depression in Nigerian children under 5 that can’t be attributed to potential confounders, including body weight, maternal education, and child functional difficulties (Kanellopoulou et al. 2022; Malhotra and Sahoo 2018). It is possible the low baseline rates of breastfeeding in Sub-Saharan African countries (Issaka et al. 2017), combined with a high prevalence of childhood trauma in the region (e.g., exposure violence, poverty) (Kurtz et al. 2023; Amene et al. 2024) may magnify any beneficial effects of breastfeeding on mental wellbeing in local children (Krol and Grossmann 2018).

Gender played an important role in the relationship between breastfeeding and depression. We observed an association primarily in female children. The reason for this gender effect is unclear but may partly reflect gender differences in breastfeeding practices. However, a systematic analysis of qualitative data on breastfeeding in sub-Saharan Africa revealed no qualitative differences in how mothers breastfeed male and female children (Ejie et al. 2021). One important consideration that may not be captured in qualitative data is the *duration* of breast feeding. Research suggests breastfeeding reduces the risk of depression only in children breastfed for longer than 6 (a shorter duration of breastfeeding was not associated with any protective effect) (Huang et al. 2019). Gender may determine breastfeeding duration (e.g., early initiation) in sub-Saharan Africa, whereby mothers breastfeed female children for longer. However, evidence in this area is mixed. One study found female children were more likely to be breastfed early (Woldeamanuel 2020), while another study found the opposite (Ayalew et al. 2019). Further research is necessary to determine if gender serves as a proxy for other variables affecting breastfeeding initiation/duration, such as local ethnicity, religious beliefs, and birth order (Shimizu et al. 2023).

Use of antimalarial medicines, specifically ACT, was associated with paediatric depression, but only in male children (boys exposed to ACT were 2.3 times more likely to experience depression compared to male children administered ACT drugs). While the link between antimalarial drugs and psychiatric conditions is complex and not well understood (Nevin and Croft 2016), the current findings may depict a gender-based disparity in exposure to anti malaria drugs, whereby male children are more likely to be administered ACT, and hence experience (and express) emotional discomfort from this treatment (Annan et al. 2023). Although research suggests malaria prevalence and associated risk factors in Sub-Saharan Africa are similar for male and female children aged under 5 (Nwaneli et al. 2020; Chilot et al. 2023), survey data from thirteen sub-Saharan African countries indicates gender affects whether children receive prompt access to antimalaria drugs (albeit not ACT medicines specifically) (Shah et al. 2015). However, evidence from a survey of antimalarial drug consumption in children under 5 from five countries in sub-Saharan African found no gender effect (Hossain et al. 2023a). Another possible explanation is that male children are simply more susceptible to depression, and hence are more likely to experience emotional distress when exposed to uncomfortable antimalarial treatment (Creek et al. 2010). However, data suggests gender differences in depression amongst children is perfunctory (Salk et al. 2017), and more research is needed in this area.

Poverty and childhood trauma might be important in understanding the link between ACT and depression in male children. Depression is more prevalent among African children exposed to violence (Jorns-Presentati et al. 2021) or exploitative child labour (Ibrahim et al. 2019). Furthermore, a survey of adverse childhood experiences in five sub-Saharan African countries found that male children are more likely than their female counterparts to witness and experience physical violence (Amene et al. 2024). Thus, the former group may already be more mentally distressed, from violent childhood traumas, prior to been administered antimalarial drugs. The medication may simply accentuate this pre-existing psychiatric vulnerability, manifesting as depression (Nevin and Croft 2016). However, evidence for this view is weak. For example, a survey of the prevalence of emotional abuse experienced by children from Sub-Saharan Africa observed no gender effect (Kurtz et al. 2023). Furthermore, research with Nigerian children specifically has found no gender differences in exposure to violence, physical abuse, and other traumatic experiences, compared their female counterparts (Asagba et al. 2021). Moreover, data suggests girls are more likely to be victims of sexual abuse/trauma (Ibrahim et al. 2019; Amene et al. 2024). Overall, further research is necessary to better understand how gender affects the association between antimalarial treatment and depression in sub-Saharan African children.

## Paper 2: the role of antimicrobial medicines

### Introduction

Use of antibiotics to treat febrile illnesses like respiratory tract infections and diarrhoeal diseases in young children is common in sub-Saharan Africa, especially in Nigeria (Auta et al. 2019). DHS data on antibiotic treatments in children under 5, from 45 countries (438 140 child-observations), showed that 38.4% (95% CI, 37.9 – 38.8%) of children with febrile symptoms received antibiotics (Levine et al. 2022). Other data from LMICs shows antibiotics were prescribed to 80·5% of children diagnosed with respiratory illness, 50·1% with diarrhoea, and 28·3% with malaria. The mean number of antibiotic prescriptions issued to children between birth and age 5 years across eight LMICs was 24·5 (95% CI 22·6–26·7), ranging from 7·1 (6·3–7·9) in Senegal to 59·1 (54·1–64·6) in Uganda (Fink et al. 2020).

In Nigeria specifically, total antibiotic exposure in the first 5 years of life was 9.6 (95% CI 8.6–10.6) with formal health services being the main source of antibiotics in most cases (Levine et al. 2022). A study by Hossain et al. estimated that 4.3% of Nigerian children with fever were exposed to antibiotic use (Hossain et al. 2023b). Nigerian children aged under 5 years are more likely to be exposed to antibiotics by their mothers (Efunshile et al. 2019). An analysis of 450 out-patient case notes for under 5 years children from two Nigerian maternity hospitals, and medical records of 3700 under-five children treated at a third hospital, found that the majority (72%) of mothers administered antibiotics to their child, with only about half (48.5%) prescribed by a physician (Adisa et al. 2018). A meta-analysis of data from 30 countries in sub-Saharan Africa revealed that Nigeria has one of the highest rates of antibiotic usage in West African children under 5 years (Auta et al. 2019).

Antibiotic usage has also been associated with an increased risk of psychiatric disorders, including depression (Lurie et al. 2015; Lavebratt et al. 2019; Prichett et al. 2022), through complex bidirectional biological interactions between gut bacteria and the brain (‘brain–gut–microbiota axis’) (Dinan and Dinan 2022). However, most research in this area has focused on adult, adolescent, or mixed populations, and moreover isn’t specific to sub-Saharan Africa (Lurie et al. 2015; Prichett et al. 2022). Some limited data suggests antibiotics exposure may contribute to depression in children aged a under 5. For example, a population-based longitudinal study of 1 million births in Finland, from 1996 to 2012, followed up through 2014, found that antibiotics exposure in utero and during the first two postnatal years was associated with a slightly higher risk of psychiatric dysfunction, including mood disorders (e.g., depression), after adjusting for pregnancy- and birth-related covariates. Antibiotic exposure during pregnancy (trimesters 1 to 3) was associated with a 14–15% increased risk of offspring psychopathology. Antibiotics usage after 2 years age was also related to a slightly increased risk of psychiatric dysfunction (Lavebratt et al. 2019).

### Objective

Paper 2 explores the role of antibiotics in suspected depression amongst children aged under 5. We hypothesised that children suspected to have depression are less likely to have consumed antibiotic medication, in line with previous work.

### Method

#### Sample

Data on antibiotic medicinal use was extracted from the original sample of 31,103 children aged under 5 years (see Paper 1).

#### Measures

##### Exposure to antibiotics

Use of antibiotics was also assessed via the multiple-choice questions in the ‘Care of Illness’ section of the survey (MICS 2022). As previously stated, this section assessed the type and/or source of advice or treatment provided in response to childhood illnesses during the past fortnight, notably diarrhoea, fever, and cough. Antibiotic usage was assessed via the same questions used to gauge antimalarial drug use. Four types of antibiotics were identified: amoxicillin (coded A), cotrimoxazole (coded M), other antibiotic pill/syrup (coded N), and other antibiotic injection/IV (coded O). Given their unspecified labels, the two latter antibiotic categories were excluded from data analysis. Finally, use of home remedies and herbal medicines was also recorded as a separate medicinal category (coded Q). Exposure to each medicine type was treated as a separate dichotomous variable – used (coded 1) or not used (coded 0).

##### Confounding factors

Accounting for childhood fever and cough was essential since these symptoms are associated with depression symptoms in children (Chaplin et al. 2020), and hence may precipitate antibiotics exposure, potentially confounding the relationship between antimicrobial use and childhood depression.

## Results

### Descriptive data

Depressed children were more like than non-depressed participants to be administered cotrimoxazole (1% versus 0.6%, respectively), (χ^2^ (1, *N* = 16720) = 6.495, *p* < 0.01). There were no group differences in the use of amoxicillin or herbal antibiotics across gender, age, and ethnicity/tribe. Only a small minority of children (≤ 4.8%) had been exposed to antibiotics.

### Predicting childhood depression

Logistic regression estimates and overall model parameters are displayed in Tables 2, 3 and 4. Exposure to antibiotics failed to predict childhood depression across the whole sample (Table 2), and separately for males (Table 3) and females (Table 4).

#### Robustness checks

Our multiple robustness checks (see Paper 1) confirmed the original analyses. Overall, antibiotic exposure failed to predict depression, with one exception: when we re-ran the analyses based on medical symptoms (cough, fever), use of the antibiotic cotrimoxazole significantly predicted depression in non-febrile children (AOR = 6.023, 95% CI [1.357– 26.721]).

## Discussion

We found little evidence linking antibiotics and childhood depression, in contrast with previous research implicating antibiotic usage with an increased risk of mental disorders, including depression (Lurie et al. 2015; Lavebratt et al. 2019; Prichett et al. 2022). However, previous work in this area has been largely based on adult, adolescent, or mixed populations, and was not specific to sub-Saharan Africa (Lurie et al. 2015; Prichett et al. 2022). The observed association between antibiotics and depression solely in non-febrile children may denote a tendency for antimicrobials to be administered to children not diagnosed with malaria (i.e., not feverish), thereby increasing their risk of depression from antibiotics exposure (Dinan and Dinan 2022). This view is supported by data from LMICs showing that antibiotics are less likely to be administered to children diagnosed with malaria (28.3%), compared to other medical conditions, such as respiratory illness (80·5%), and diarrhoea (50.1%) (Fink et al. 2020). Further research is needed to better understand how and why antibiotics may contribute to depression in non-febrile children under 5.

## General discussion

This population-based study helps address the lack of data on paediatric depression and its correlates, in young children from sub-Sharan Africa (Tomlinson and Morgan 2015). Analysis of data from 30,804 Nigerian children aged under 5 years showed that those suspected to have depression were more likely to have received antimalarial medicines, specifically ACT and artesunate injections, and not been breastfed. The association between ACT exposure and depression was much stronger in male children, whereas the relationship of breastfeeding with depression applied primarily to female children. Crucially, these associations remained significant despite adjusting for important covariates, albeit multiple robustness checks suggest contextual factors including mother’s education, geographical area, and household wealth, and medical symptoms (fever, cough) have an important moderating effect. Overall, this evidence supports previous research linking antimalarial medication to psychiatric conditions, including depression (Bitta et al. 2017; Nevin and Croft 2016; Maxwell et al. 2015). The data also implicates breastfeeding (Bugaeva et al. 2023). Contrary to previous work with older demographics (Lurie et al. 2015; Lavebratt et al. 2019; Prichett et al. 2022), we found limited evidence linking antibiotics with depression in children under 5.

### Limitations

This study has several limitations. Firstly, diagnosis of childhood depression was based on mother’s or caretaker’s subjective judgements, in the form of a single-item question. While such parental feedback can be useful for understanding psychopathology in children (Lewis et al. 2012), it may not accurately capture a child’s emotional state (Caqueo-Urízar et al. 2022), and could be affected by the parents own mental health (Kamis 2021). Paediatric depression is a complex disorder that is difficult to diagnose, and parental/caregiver judgements may fail to capture this intricacy (Merten et al. 2017). Symptoms vary with age, level of development, and comorbidities, for example (Patra 2019). While depression has been diagnosed in children as young as 3 years, the symptoms may not meet clinical diagnostic criteria (Mullen 2018). Proper diagnosis often requires clinical interviews with both parental and child, conducted on an ongoing basis, and requiring the use of pictorial instruments and depression ratings scales (Patra 2019).

Secondly, robustness checks suggest the findings may vary depending on the type of breastfeeding measure used, and group differences in socio-demographic factors (e.g., age, geographical area, wealth index, mother’s education, medical symptoms). However, these inconsistencies did not dramatically alter the recurring pattern whereby antimalarial medicinal use, particularly ACT treatment, and breastfeeding predicted childhood depression. The data was also generally unaffected by the method used to manage missing data.

It is notable that current breastfeeding failed to predict depression, perhaps reflecting the greater challenge of identifying ‘sadness’ in very young children (Patra 2019). Furthermore, breastfeeding duration not assessed. This is a potentially important metric that may moderate the association between breastfeeding and paediatric depression (children breastfed for > 6 months have a reduced risk of depression) (Huang et al. 2019). Since breastfeeding rates in West and Central African countries fall below the WHO’s recommended 50% (Issaka et al. 2017), breastfeeding duration might play a particularly important role in child health across the region, significantly impacting the odds of childhood depression.

Thirdly, we did not control for several potential confounding factors implicated in childhood depression including family genetics, maternal depression, childhood maltreatment, and cognitive vulnerability (Lima et al. 2013; Malhotra and Sahoo 2018). Severe childhood trauma (e.g., experiencing violence) in particular is highly prevalent in sub-Saharan Africa (Amene et al. 2024; Kurtz et al. 2023), and has been strongly associated with mood disorders in children (Jorns-Presentati et al. 2021). Further research is therefore needed to determine how this covariate interacts with medication use and breastfeeding in predicting paediatric depression.

Despite these constraints, this study has several strengths. Firstly, to the best of our knowledge, this is the first ever investigation to demonstrate the role of antimalarial treatment, and breastfeeding, in the mental health of Nigerian children aged under 5 years. There is currently little or no published data on mood disorders and its correlates in very young children from this geographical region (Tomlinson and Morgan 2015). Most research in this area has focused on adolescents, adults, or mixed populations (Gbadamosi et al. 2022; Yohani et al. 2023; Sequeira et al. 2022; Partap et al. 2023; Aluh et al. 2018; Jorns-Presentati et al. 2021; Mabrouk et al. 2022; Owen et al. 2016; Cortina et al. 2012; Sherr et al. 2020; Mokwena et al. 2023). Secondly, we analysed data from a very large sample, providing smaller margins of error in adjusted odds ratio estimates, and hence generating more robust and reliable results. Third, we did control for several important covariates implicated in paediatric depression, including body weight (Kanellopoulou et al. 2022), and socio-demographic factors, such as maternal education, and households’ cumulative living standards (wealth index) (Malhotra and Sahoo 2018).

## Conclusions

This is the first population-based study to examine the role of breastfeeding and medicinal use, in suspected depression amongst children aged under 5, from sub-Sharan Africa. Childhood depression was associated with exposure to antimalarial treatment, specifically ACT and artesunate injections, and lack of breastfeeding. Gender was an important moderating factor: treatment with antimalarial medicines may be an independent risk factor for paediatric depression primarily in male children, whereas the association between breast feeding and depression was stronger in female children. How breastfeeding was assessed, and variations in mother’s educational level, geographical area, household wealth, and medical symptoms (e.g., fever), were also important contextual factors. Given that children in sub-Saharan Africa often face mental health challenges aggravated by chronic poverty, and childhood trauma, exposure to antimalaria treatment and inadequate breastfeeding may help identify Nigerian children under 5 who are susceptible to depression.

## Data Availability

Due to third party rights, other legal and ethical reasons, and the nature of data gathered, MICS data may not be redistributed or shared publicly, in any form (MICS, 2022). Access is restricted and facilitated directly by UNICEF/MICS (MICS, 2024). All MICS-related data and documentation can be viewed at http://www.childinfo.org/mics including questionnaires, manuals, data processing and tabulation plans as well as national reports, datasets and contact information. Furthermore, results from the surveys are made available in DevInfo, a powerful database designed to monitor progress towards the Millennium Development Goals. Access to MICS data requires registration at mics@unicef.org. For further information please contact the Global MICS Coordinator, Strategic Information Section, Division of Policy and Planning, UNICEF, 3 U.N. Plaza, New York, NY 10017, USA. Tel: 212 303 7982.

## Abbreviations

ACT: Artemisinin Combination Therapy
AOR: Adjusted Odds Ratio
BMGF: Bill and Melinda Gates Foundation
CFD: Child’s Functional Difficulties
CNS: Central Nervous System
EM: Expectation Maximisation
FMoH: Federal Ministry of Health (Nigeria)
GAVI: the Vaccine Alliance
LMIC: Low and middle-income countries
MICS: Multiple Indicator Cluster Survey
NBS: National Bureau of Statistics
NICS: National Immunization Coverage Survey
NMEP: National Malaria Elimination Programme
OR: Odds Ratio
UNICEF: United Nations Children’s Fund
WHO: World Health Organisation
